# Recommendations for assessing commutability of a replacement batch of a secondary calibrator certified reference material

**DOI:** 10.1016/j.cca.2024.120097

**Published:** 2025-02-01

**Authors:** Liesbet Deprez, Jesper V. Johansen, Thomas Keller, Jeffrey Budd, Neil Greenberg, Cas Weykamp, Sverre Sandberg, Mauro Panteghini, Ferruccio Ceriotti, Elizabeth Barczak, Robert Rej, Pernille Kjeilen Fauskanger, Finlay MacKenzie, Johanna E. Camara, Alicia N. Lyle, W.Greg Miller, Vincent Delatour

**Affiliations:** a European Commission, Joint Research Centre (JRC), Geel, Belgium; b Radiometer Medical ApS, Copenhagen, Denmark; c ACOMED Statistic, Leipzig, Germany; d Jeff Budd Consulting, St. Paul, MN, United States; e Neil Greenberg Consulting, LLC, Rochester, NY, United States; f Queen Beatrix Hospital, Winterswijk, the Netherlands; g Norwegian Organization for Quality Improvement of Laboratory Examinations (Noklus), Haraldsplass Deaconess Hospital, Bergen, Norway; h Norwegian Porphyria Centre, Department of Medical Biochemistry and Pharmacology, Haukeland University Hospital, Bergen, Norway; i Department of Global Public Health and Primary Care, University of Bergen, Bergen, Norway; j Department of Laboratory Medicine, Ludwik Rydygier Collegium Medicum in Bydgoszcz, Nicolaus Copernicus University in Torun, Torun, Poland; k Fondazione IRCCS Ca’ Granda Ospedale Maggiore Policlinico, Milan, Italy; l Siemens Healthineers, Newark, United States; m Department of Biomedical Sciences, School of Public Health, University at Albany, State University of New York, Albany, NY, United States; n Department of Mathematics, University of Bergen, Bergen, Norway; o Birmingham Quality/UK NEQAS, University Hospitals Birmingham NHS Foundation Trust, Birmingham, United Kingdom; p National Institute of Standards and Technology, Gaithersburg, MD, United States; q Centers for Disease Control and Prevention, Atlanta, GA, United States; r Department of Pathology, Virginia Commonwealth University, Richmond, VA, United States; s Laboratoire national de métrologie et d’essais, Paris, France

**Keywords:** Certified Reference Material, Commutability, Metrological Traceability, Replacement Batch

## Abstract

Commutable secondary certified reference materials (CRMs) play an essential role in the calibration hierarchy of many in-vitro diagnostic measurement procedures used in the medical laboratory. Therefore, sustainable availability of these CRMs is crucial to guarantee the long-term equivalence of results obtained for the clinical samples. The IFCC Working Group on Commutability in Metrological Traceability (WG-CMT) has published several recommendations for assessing the commutability of secondary calibrator CRMs. Performing a full commutability study according to these recommendations may present significant demands on the resources of CRM producers.

This report provides recommendations for performing commutability equivalence assessments between existing CRMs of proven commutability and replacement batches of those CRMs. The approach evaluates the relationship of measurement results obtained with the relevant measurement procedures for the replacement batch versus the existing CRM batch. If this relationship is the same, the commutability properties of the replacement batch are considered equivalent to those of the existing CRM batch. Since the existing batch has a suitable commutability, the commutability of the replacement batch is also declared fit for purpose. Because this commutability equivalence assessment involves certain risks, a small number of representative clinical samples are included as safeguards.

There are several prerequisites for performing the commutability equivalence assessment and producers of secondary CRMs will probably need to implement improvements before using this approach. However, once the improvements are implemented, the commutability equivalence assessment approach will significantly reduce the resources needed to maintain the supply of CRMs.

## Highlights

• Commutable CRMs are critical for equivalent laboratory results among procedures.
• Resource-intense commutability studies may limit replacement batch CRM supply.
• Equivalence assessment is efficient; uses old and new CRM and 2–3 clinical samples.
• Several conditions must be met before applying this approach.
• If conditions are met, this approach may improve CRM replacement batch availability.

## Nomenclature

CRM: certified reference material
CS: clinical sample
CV: coefficient of variance
FIN: further investigations needed
IVD: in-vitro diagnostic
JCTLM: Joint Committee for Traceability in Laboratory Medicine
MANCB: maximum allowable noncommutability bias
MP: measurement procedure
nCRM: new CRM
oCRM: original CRM
SD: standard deviation
TOST: two one sided t-test
rCS: representative clinical sample
RMP: reference measurement procedure
WG-CMT: Working Group on Commutability in Metrological Traceability

## 1 Introduction

Laboratory tests play an important role in many medical decisions and the optimal interpretation of their results depends on agreed clinical practice decision thresholds or common reference limits. It is essential to guarantee equivalent results for the clinical samples (CSs) irrespective of the end-user in-vitro diagnostic (IVD) measurement procedure (MP) used in the medical laboratory. Equivalent results for CSs can be achieved by establishing metrological traceability of the values assigned to the calibrators used in the IVD-MPs as specified in ISO 17511:2020 [1]. Metrological traceability describes the calibration hierarchy as an unbroken chain of value assignments between the measurement results of the CSs up to the highest available component in the calibration hierarchy. Commutable secondary certified reference materials (CRMs) play an essential role in the calibration hierarchy as they can be used as calibrators (position m.3 in ISO 17511:2020) for the value assignment procedures for the manufacturer’s internal calibrators and/or the end-user calibrators for the IVD-MPs intended to measure the same measurand.

Commutability with CSs is an essential requirement for secondary calibrator CRMs. The IFCC Working Group on Commutability in Metrological Traceability (WG-CMT) has published recommendations for assessing commutability [2], [3], [4], [5]. Performing a full commutability assessment according to these recommendations present significant demands on time, people, facilities, and budgets for CRM producers [6], [7]. Working with limited resources and competing demands, and in view of the potential costs of full commutability assessments, CRM producers may choose to delay or cancel production of replacement batches of an existing CRM when available inventory becomes depleted. Additionally, when a CRM producer chooses to dedicate substantial resources to production and commutability assessment of replacement batches of existing CRMs, budget and resource constraints may lead to delay or cancellation of projects to develop new CRMs for standardization of other measurands.

An assumption cannot be made that a replacement batch of a CRM will have the same commutability properties as its predecessor just because it underwent similar production processes. Secondary calibrator CRMs are frequently based on biologically sourced materials that are subject to substantial variation from batch to batch. Process variations in manufacturing steps can also contribute to significant variation in the performance characteristics of new CRM batches. Therefore, an assessment of commutability of the replacement batch is required. When the existing CRM batch has successfully completed a full commutability assessment and consistently demonstrated adequate commutability, a simplified commutability assessment procedure may be applicable for a replacement batch.

The present recommendations for a commutability equivalence assessment of qualified new batches of existing secondary CRMs are intended to encourage CRM producers to manufacture replacement batches and nominate those to the Joint Committee for Traceability in Laboratory Medicine (JCTLM) for credentialing and listing in the JCTLM database [8], [9]. Availability of replacement batches helps to ensure ongoing availability of critical CRMs necessary to support manufacturing processes for IVD-MPs, and sustain a viable calibration infrastructure over the long-term to benefit all stakeholders, including medical laboratories, patients, healthcare providers, CRM producers, and manufacturers of IVD-MPs.

## 2 Concept of a commutability equivalence assessment

A commutability equivalence assessment compares a candidate replacement batch, referred to as new CRM (nCRM) to the original CRM batch (oCRM). This approach is based on the assumption that: (a) the oCRM has acceptable commutability with CSs; (b) the MPs in current use are substantially the same as those included in the original commutability assessment; and (c) the nCRM is produced in the same manner as the oCRM.

The proposed approach measures samples from the nCRM and the oCRM using the relevant MPs. For each MP, the difference between results measured for nCRM and for oCRM is assessed. If this difference is the same, within a criterion, for all MPs included in the assessment, then the nCRM and oCRM can be considered to have equivalent commutability properties for each MP. As the oCRM has a suitable commutability (prerequisite for performing the commutability equivalence assessment), the commutability of the nCRM can be declared fit for purpose.

While the purpose of a commutability equivalence assessment is to demonstrate an equivalent commutability of an nCRM with that of an oCRM, verifying that commutability of the nCRM is fit for purpose is the overall goal. Comparing commutability of the nCRM against the oCRM has risks. The commutability of the oCRM might no longer be adequate due to i) instability of the oCRM and/or ii) undetected changes in the performance of the MPs. Consequently, a small number of representative clinical samples (rCSs), single donation CSs and/or qualified pools, are included in the commutability equivalence assessment to support the commutability of the nCRM and to identify possible problems with the commutability stability of the oCRM.

## 3 Requirements for conducting a commutability equivalence assessment

**Fig. 1:**
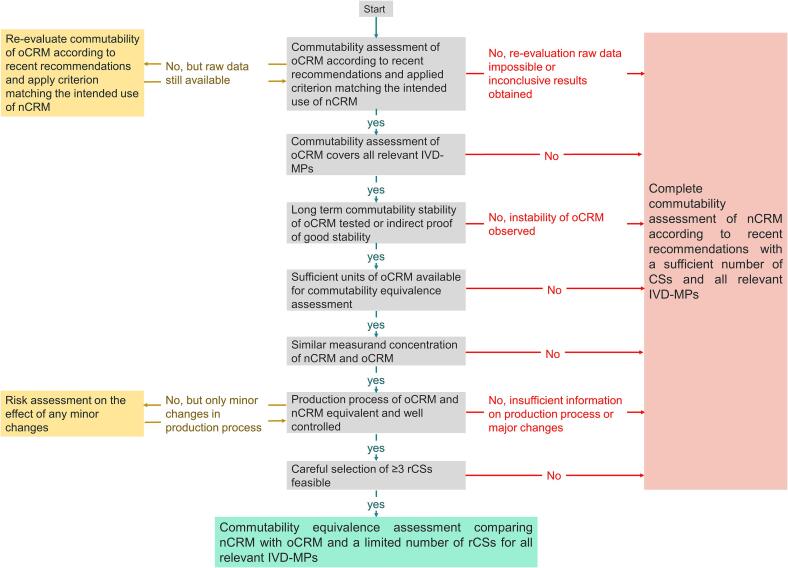
Decision tree for conducting a commutability equivalence assessment CSs: clinical samples, IVD-MPs: in-vitro diagnostic measurement procedures, nCRM: new CRM batch, oCRM: original CRM batch, rCSs: representative clinical samples.

Requirements for using a commutability equivalence assessment are summarized in Fig. 1 and in a checklist provided in Supplementary material section 2.

### 3.1 Requirements for the oCRM

#### 3.1.1 Commutability of the oCRM

The most important prerequisite for conducting a commutability equivalence assessment is the design and outcome of the original full commutability assessment on the oCRM. The reliability of this commutability assessment is crucial and mainly depends on two aspects.

First, the original full commutability assessment of the oCRM should have been designed and evaluated according to the recent recommendations [2], [3], [4], [5], [10] and with a criterion [5] matching the intended use of the nCRM. However, CRMs often have a long shelf life (>10 years) and many of the CRMs available at the time of this recommendation for commutability equivalence assessment were produced before publication of the IFCC recommendations. The commutability assessment of the oCRM might have been performed with other approaches such as CLSI EP14 [11] or EP30-A [12]. These approaches have certain limitations as they do not quantify the uncertainty associated with the commutability assessment, and the prediction intervals might be too wide in cases with large differences in non-selectivity between the MPs in relation to the clinical application requirements [2], [13], [14].

A risk-based approach should be used by CRM producers when assessing whether the commutability assessment for the oCRM was acceptable. Risk assessment should be performed following the requirements of ISO 13485 [15], especially in consideration that risk assessment should focus on the safety and performance of the nCRM. Detailed guidance on the application of various risk management tools is available [16], [17].

If the original commutability study was not conducted according to the recent recommendations [2], [3], [4], [5], [10], it might be necessary to reanalyze the data from the original study with a suitable commutability criterion and to confirm that the number of replicate measurements performed on the CRM and the CSs were sufficient for an acceptable estimate of the uncertainty associated with the commutability assessment. If the old raw data are no longer available, or if reanalysis leads to unacceptable uncertainties or raises concerns about commutability of the oCRM, a new complete commutability assessment is needed for the nCRM following the newer recommendations.

Second, the MPs involved in the original commutability study must be adequately representative of those in current use in medical laboratories and their performance should be basically unchanged since the assessment of the oCRM. The commutability properties are assumed to remain the same over many reagent lots and other maintenance to a measuring system. Examples of changes in a MP that could alter its performance characteristics (including its selectivity) include: reformulation of a reagent; different origin of active biomolecules (e.g. antibodies or enzymes); changes in pre-treatment conditions such as extraction or chromatography separation; adapting reagents to a new instrument platform; using a different sample type; etc. For some common measurands, the available IVD-MPs might seem unchanged for several years, however, IVD-MP adaptations are often not known by the CRM producer.

If a substantial number of IVD-MPs currently in use by medical laboratories were not represented in the commutability assessment of the oCRM, then a complete commutability assessment for the nCRM is needed.

A commutability assessment conducted by others after release of the oCRM should carry the same weight as a commutability assessment conducted by the CRM producer, if an acceptable approach was used for the commutability assessment.

#### 3.1.2 Stability of the commutability of the oCRM

The commutability characteristics of a CRM can change over time. The stability of the oCRM must be adequate to ensure it has maintained the same commutability properties as when the original full commutability study was conducted. Ideally, there should be proof of the stability of the commutability properties of the oCRM such as the results of regular stability checks performed with different MPs [18]. However, in most scenarios no information will be available about the stability of commutability characteristics of an oCRM. In these cases, supportive information on stability can be obtained within the commutability equivalence assessment by comparing the biases of the oCRM and the nCRM to the biases of the rCSs. If a nCRM is produced because of proven or suspected instability of the oCRM, a full commutability study must be conducted for the nCRM.

#### 3.1.3 Availability of the oCRM

A commutability equivalence assessment requires a substantial number of vials of the oCRM to be available as the commutability equivalence assessment still needs to be performed on a number of relevant MPs. Also, given that commutability will be evaluated using only the measurement results obtained on the oCRM and the nCRM, the number of replicate measurements required to reduce uncertainties and minimize the frequency of indeterminate results might be higher than in a full commutability study. The assessment should therefore start before the oCRM batch is depleted.

### 3.2 Requirements for the nCRM

The commutability equivalence assessment is suitable to compare an nCRM to an oCRM batch only when the nCRM is produced to be equivalent to the oCRM. Equivalent means that at least two conditions must be fulfilled: i) the measurand concentration of the nCRM must be reasonably close to that of the oCRM, and ii) the nCRM is intended and expected to have the same commutability properties as the oCRM, which means that production process and raw materials are not substantially different.

#### 3.2.1 Measurand concentrations in the nCRM

The measurand concentrations in the nCRM and oCRM must be sufficiently close that the imprecision of measuring the nCRM and oCRM is the same (in concentration units, standard deviation (SD), or relative to the concentration, coefficient of variance (CV)) for all MPs. When the concentrations are too different and similar imprecision is not achieved, then a full commutability assessment is needed.

When a CRM consists of a series of materials (e.g. various concentration levels of the measurand), or when CRM dilutions are expected to be prepared for its intended use, the above still applies. If each nCRM level matches an oCRM level, the commutability equivalence assessment can be used. Note that different levels should be compared in separate studies. If the levels of the nCRM do not match the levels of the oCRM, a full commutability study will be needed.

#### 3.2.2 Formulation and production process of the nCRM

The nCRM is expected to be produced with the intention of replicating the production of the oCRM. To maximize the likelihood that commutability of the nCRM and the oCRM will be equivalent, the specifications for preparation of an nCRM should be practically identical to those of the oCRM as described in ISO 33405:2024 [19]. The CRM producer must have sufficient knowledge of the production specifications of the oCRM and identify the crucial steps within the manufacturing procedure potentially affecting the commutability of the CRM (e.g. source of raw materials, spiking with exogenous compounds, lyophilization). By performing a risk assessment of the planned adaptations in the production process and by specifying the required quality control checks the producer can ensure an equivalent preparation. Some important considerations are described hereafter.

Secondary commutable CRMs are frequently based on biological materials obtained from human donors and the constitution of the raw materials can be affected by many factors. The biological materials as well as potential interfering substances can be subject to inter-individual differences among donors. Changes in the disease status, ethnicity or sex distribution of the donors should be evaluated for potential impact on the commutability characteristics of the nCRM.

Modification in the collection and subsequent handling of the biological materials can also have a large impact. Strict and detailed collection protocols (e.g. the updated CLSI C37 [20]) are recommended especially if the collection center has changed compared to the oCRM or if multiple collection centers are used.

After collecting the biological materials, additional manipulations are often performed by the CRM producer like pooling, spiking, filling of the CRM units, and lyophilization or freezing. The equipment used during this manufacturing process is likely to change over time. The manufacturer should gather information about the effect of certain process parameters, like freezing speed, freeze thaw cycles and lyophilization conditions, on the measurand. Changes in the nature and amount of added exogenous substances, like stabilizers, should also be considered.

### 3.3 Representative clinical samples (rCSs)

The commutability equivalence assessment must include a minimum of three rCSs. These samples are important safeguards providing supportive information, especially to indicate that commutability of the oCRM did not change over time. The measurand concentrations in the rCSs must be close to the measurand concentrations of the nCRM being evaluated. The samples should either be native single donation CSs or pools of CSs. As a general rule, we recommend including two single donation CSs and one pooled sample. The specific number of CSs and pools can be adjusted based on practical concerns, knowledge of prevalence of interferents in CS, and knowledge of commutability of pools. Selection of CSs and the potential to use CS pools are described in a previous recommendation [2]. If good rCSs cannot be selected, conducting a full commutability study with a large number of pooled CSs may be desirable because any effects of unknown interfering substances are reduced in this study design.

### 3.4 Consecutive commutability equivalence assessments

Consecutive commutability equivalence assessments should not be performed to assess the commutability of several sequential nCRM batches in which the nCRM batch is only compared to its immediate predecessor. Accumulation of small noncommutability biases that are acceptable in an individual assessment might cause unacceptable noncommutability bias to be undetected after several replacement batches. A commutability equivalence assessment should always include samples designated as oCRM that had an acceptable full commutability assessment to prevent accumulation of noncommutability biases.

## 4 Study design of the commutability equivalence assessment

### 4.1 Establishing the maximum allowable noncommutability bias criterion

The commutability criterion, i.e., the maximum allowable noncommutability bias (MANCB), directly affects the number of replicate measurements that should be performed in the study. This criterion should be established before the study is designed, following the principles described by the IFCC WG on CMT [5]. Using the same MANCB as that used in the original commutability assessment for the oCRM is generally considered acceptable assuming it is consistent with the principles described in [5].

### 4.2 Mps included in the study

The IVD-MPs included in the commutability equivalence assessment should be adequately representative of those in current use in medical laboratories. A reference MP (RMP) should be included when available and practically possible. Realistic estimates of the repeatability of each MP are required and this information may be obtained from the manufacturer’s instructions for use, from External Quality Assessment scheme reports, or from imprecisions estimated from the commutability study of the oCRM.

Both nCRM, oCRM and the rCSs must be measured with each MP using the same reagent and calibrator lots and preferably within a single run. The measurement sequence of the replicates of the nCRM and the oCRM should be in an interleaved manner (e.g., run order: nCRM_1_, oCRM_1_, nCRM_2_, oCRM_2_, …), while the replicates of the rCSs are equally distributed over the measurement sequence. In this way, variance components from instrument, reagent lot and run, as well as any possible influence of run sequence on the results, are eliminated.

### 4.3 Number of replicates for CRMs

Both the nCRM and the oCRM should be measured in a sufficient number of replicates to allow a robust conclusion regarding the equivalence of their commutability. As the study should be conducted within a single measurement run, only repeatability will affect the uncertainty of the difference in bias estimate. Therefore, the number of replicate measurements will depend on the repeatability of the MPs and the commutability criterion. If it is not possible to conduct the measurements within a single run, the combined effect of repeatability and between-run imprecision must be considered for the affected MPs. The number of measurements needed (*n_MP1_*) to achieve a given statistical power (probability of obtaining a conclusion of “commutable” for nCRM that truly has commutability properties equivalent to those of the oCRM) is given in Table 1 for the most precise MP, i.e. the MP with the lowest imprecision (*s_MP1_*).

**Table 1:** Minimum numbers of measurements of CRM (*n_MP1_*), depending on the magnitude of the SD for the MP with the lowest repeatability imprecision (*s_MP1_*) relative to the MANCB, needed to achieve 80 %, 90 % or 95 % power using the two one sided t-test (TOST) approach at α-level 0.05 as described previously [5].

	**Power**		
	**80 %**	**90 %**	**95 %**
*s_MP1_***/MANCB**	**n_MP1_**	**n_MP1_**	**n_MP1_**
0.33	5	6	7
0.4	7	8	10
0.5	10	12	14
0.6	14	17	20
0.7	18	22	27
0.8	23	29	34
0.9	29	36	43
1.0	35	44	53
1.1	43	54	64
1.25	55	69	82

The table is adapted from Table S2.2 from the CRM criterion paper [5]. The changes are:

• Imprecision is the same for both materials (here nCRM:oCRM, in [5] CS:CRM). This is because the commutability equivalence assessment does not incorporate between-CS imprecision. Hence, the ratio is 1:1.

• Due to the previous point, sampling is the same for both materials (here nCRM:oCRM, in [5] CS:CRM). Hence, the ratio is 1:1.

• Expected difference (Δ) is 0 because the nCRM is designed to be as identical to the oCRM as possible.

Additionally, more levels of *s_MP1_*/MANCB are added, but they are calculated the same as described in [5].

The number of measurements needed (*n_MPX_*) for the other MPs (MPx) included in the study can be calculated from the imprecisions ( *s_MPX_*) of the respective MPs, using Eq. (1).

(1) $$n_{MPX}=n_{MP1}\frac{s_{MPX}}{s_{MP1}}$$

The calculated sample sizes are the minimum sample sizes needed to achieve the desired power. This minimum sample size, based on Eq. (1), may be different for every MP included in the study. Having different sample sizes may not be practical and it is acceptable to round up all sample sizes to match the sample size needed for the MP with the highest imprecision. If the MPs included in the study have very different imprecisions, it may be practical to generate groups of MPs with similar imprecision and use the highest minimum sample size for all MPs in each group. Table 1 shows minimum numbers of measurements of CRM needed to achieve 80 %, 90 % or 95 % power as described previously [5].

### 4.4 Number of replicates for rCSs

The rCSs are measured with 5 to 10 replicates each. Fewer measurements are required for rCSs since their use is not to prove commutability but merely to check for severe issues with the commutability stability of the oCRM or commutability equivalence of the nCRM, with one or more MPs, and/or with the study design. The exact number of replicates depends on the available amount of each rCS, the cost and effort needed to perform more measurements, and the imprecision of the measuring systems. If the minimum of five replicates cannot be met due to sample volume limitations or other technical concerns, then the impact of a smaller number of replicates (e.g. 3 or 4) should be considered (preferably according to a risk assessment). Alternatively, using additional CS pools in place of one or both single donation CSs may be considered. Such modifications to the test design should be documented and justified in the study report.

When the ratio *s_MPX_*/MANCB is larger for a particular MP, similar to the choice of the number of replicates for CRMs, increasing the number of rCS replicates for this MP may be desirable to ensure that the rCSs are effective safeguards for a confirmatory assessment.

## 5 Statistical analysis and uncertainty calculations

### 5.1 Model

The approach for nCRM and oCRM comparison is based on the approach described in the Working Group Recommendations for Assessing Commutability Part 2: Using the Difference in Bias between a Reference Material and Clinical Samples [3].

The experimental design considers the comparison of results, $x_{MP1}$ and $x_{MP2}$, obtained by two MPs (MP1 and MP2) for a given material with true concentration μ. In a typical experimental design, n replicates of each CRM (new and original) are measured in a single run with each of the MPs. A model for the bias between single determinations of a specified CRM is:

(2) $$b_{\mathrm{CRM}}=x_{MP2}-x_{MP1}+e_{x_{MP1}}+e_{x_{MP2}}$$

where:

*x_MP1_* is the mean value for MP1.

*x_MP2_* is the mean value for MP2.

$b_{\mathrm{CRM}}$ is the bias between the two MPs for a CRM.

$e_{x_{MP1}}$ is a within-run component of variation for MP1.

$e_{x_{MP2}}$ is a within-run component of variation for MP2.

The terms $e_{x_{MP1}}$ and $e_{x_{MP2}}$ are the within-run components of random variation with standard deviations $\sigma \left(e_{x_{MP1}}\right)$ and $\sigma \left(e_{x_{MP2}}\right)$. The protocol assumes that $e_{x_{MP1}}$ and $e_{x_{MP2}}$ are independent, i.e. only affected by random effects specific to each MP (i.e. no sources of error affect both MPs simultaneously nor the material being analyzed). This model applies to both nCRM and oCRM and to the rCSs. If both CRMs are similar in composition and measurand concentration to rCSs, $\sigma \left(e_{x_{MP1}}\right)$ and $\sigma \left(e_{x_{MP2}}\right)$ are assumed to be identical for both CRMs and all rCSs.

The difference in bias between nCRM and oCRM is:

(3) $$d=b_{\mathrm{nCRM}}-b_{\mathrm{oCRM}}$$

where:

$b_{\mathrm{nCRM}}$ is the bias between results from MP1 and MP2 for the nCRM.

$b_{\mathrm{oCRM}}$ is the bias between results from MP1 and MP2 for the oCRM.

While the relevant difference calculation when an rCS is involved is:

(4) $$d=b_{\mathrm{nCRM}}-b_{\mathrm{rCS}}$$

As shown in Eq. (2), the difference in bias estimate is only affected by within-run components of random variation.

### 5.2 Calculations

The approach assumes the concentrations of both oCRM, nCRM and the rCSs are all close enough that the imprecisions are approximately the same for all samples measured by the same MP. If all samples (CRMs and rCSs) are very close in concentrations, the imprecision in measurand units (SD) with each MP can be considered approximately constant. In this situation, calculations can be performed using raw measurement results. If the concentrations of CRMs and rCSs are further apart, but close enough that the relative imprecisions (CV) of measurements with each MP can be considered approximately constant, calculations can be performed using log transformed measurement results and MANCB. The log transformed MANCB can be computed as $log(1\pm \sqrt{3}\cdot u{\text{max}}_{\mathrm{nc}})$ [21]. Concentration intervals with approximately constant SD or CV can be visually assessed using plots with absolute or relative differences plotted as a function of concentration. Data for assessing constant SD or CV may be obtained from the commutability study for the oCRM or from method comparison studies between the relevant MPs. If the measurand concentrations in the rCSs are too far from the concentrations in the nCRM and oCRMs such that approximately constant SD or CV is not met, it is necessary to collect new rCSs that meet these criteria. If the concentrations of the CRMs are too far apart that neither of these criteria are met, this commutability equivalence assessment cannot be used. In such scenarios, a full commutability assessment is required for the nCRM.

The data is analyzed using two one-sided t-tests (TOST) for equivalence. In general, the nCRM is compared to the oCRM and to each of the rCSs.

For the comparison against the oCRM the proof of safety approach is used [22], [23]. The experimental hypothesis being tested is equal to the alternate hypothesis, that the difference in bias between the nCRM and oCRM are within a predefined MANCB:

Null hypothesis: $\left|b_{\mathrm{nCRM}}-b_{\mathrm{oCRM}}\right|\;\geq MANCB$.

Alternate hypothesis: $\left|b_{\mathrm{nCRM}}-b_{\mathrm{oCRM}}\right|\;<MANCB$.

The aim is to reject the null hypothesis, thus concluding that $\left|b_{\mathrm{nCRM}}-b_{\mathrm{oCRM}}\right|\;<MANCB$.

If the nCRM is commutable with rCSs, the differences in bias between the nCRM and rCSs (each rCS evaluated separately) are within a predefined MANCB.

For the rCSs, the proof of hazard approach is used [22], [23]. The experimental hypothesis being tested is equal to the null hypothesis. The aim is to fail to reject the null hypothesis, thus concluding that it cannot be disproven that $\left|b_{\mathrm{nCRM}}-b_{\mathrm{rCS}}\right|\;\leq MANCB$. Therefore, the inequality sign is reversed compared to the inequality signs of the hypotheses for the nCRM vs. oCRM comparison.

Null hypothesis: $\left|b_{\mathrm{nCRM}}-b_{\mathrm{rCS}}\right|\;\leq MANCB$.

Alternate hypothesis: $\left|b_{\mathrm{nCRM}}-b_{\mathrm{rCS}}\right|\;>MANCB$.

The equivalence of commutability of the nCRM and oCRM is investigated by calculation of the estimated difference in bias between the MP pairs in a comparison and their associated confidence interval. The estimated biases ($\hat{b}$) are calculated for all materials and all MP pairs, as exemplified below for MP1 and MP2, and for nCRM:

(5) $${\hat{b}}_{MP1:MP2,nCRM}={\overset{¯}{x}}_{MP1,nCRM}-{\overset{¯}{x}}_{MP2,nCRM}$$

where ${\overset{¯}{x}}_{MP1,nCRM}$ and ${\overset{¯}{x}}_{MP2,nCRM}$ are the average of all measurement results for the nCRM from MP1 and MP2, respectively. The calculations according to Eq. (5) are repeated for the oCRM, for each of the rCSs and for all relevant MP pairs. The estimated difference in bias ($\hat{d}$) is then calculated according to Eq. (6) exemplified for the nCRM compared to the oCRM:

(6) $${\hat{d}}_{MP1:MP2,nCRM}={\hat{b}}_{MP1:MP2,nCRM}-{\hat{b}}_{MP1:MP2,oCRM}$$

The estimated standard uncertainty of the difference ($u\left(\hat{d}\right)$) in bias is:

(7) $$u\left(\hat{d}\right)=\sqrt{\frac{s_{MP1,nCRM}^{2}}{n_{MP1,nCRM}}+\frac{s_{MP2,nCRM}^{2}}{n_{MP2,nCRM}}+\frac{s_{MP1,oCRM}^{2}}{n_{MP1,oCRM}}+\frac{s_{MP2,oCRM}^{2}}{n_{MP2,oCRM}}}$$

where s and n are the sample standard deviations and sample sizes of all measurement results for each MP and material. Generally, the true imprecision (σ, estimated by the sample SD, s) is expected to be similar for different materials measured using the same MP, and it is recommended to use the same n for both CRMs (Table 1) and fewer measurements for rCSs.

Fewer measurements are required for rCSs since the goal is to not reject the null hypothesis for these materials. The calculations according to Eqs. (5), 6, and 7 are repeated for the nCRM compared to each rCSs, ie, replacing oCRM with rCS. This calculation is repeated for all relevant MP pairs.

To evaluate commutability, the expanded uncertainty, $U\left(\hat{d}\right)$, is needed. $U\left(\hat{d}\right)$ is obtained by multiplying $u\left(\hat{d}\right)$ by a suitable coverage factor, k. A confidence interval for $b_{\mathrm{nCRM}}-b_{\mathrm{oCRM}}$ or $b_{\mathrm{rCS}}-b_{\mathrm{oCRM}}$ is then $\hat{d}\pm U\left(\hat{d}\right)$.

A k-value for (1-2α)·100 % confidence is determined as the relevant quantile from the student's t distribution for α and degrees of freedom determined using the Welch-Satterthwaite equation [24], [25]. A *k* = 1.9 is recommended for a coverage of at least 90 % for the nCRM vs. oCRM [3] and a coverage factor of *k* = 2.3 for a coverage of at least 90 % for the rCSs vs nCRM with at least five rCS replicates. Examples of calculations are presented in Supplemental Data.

### 5.3 Drawing conclusions

**Fig. 2:**
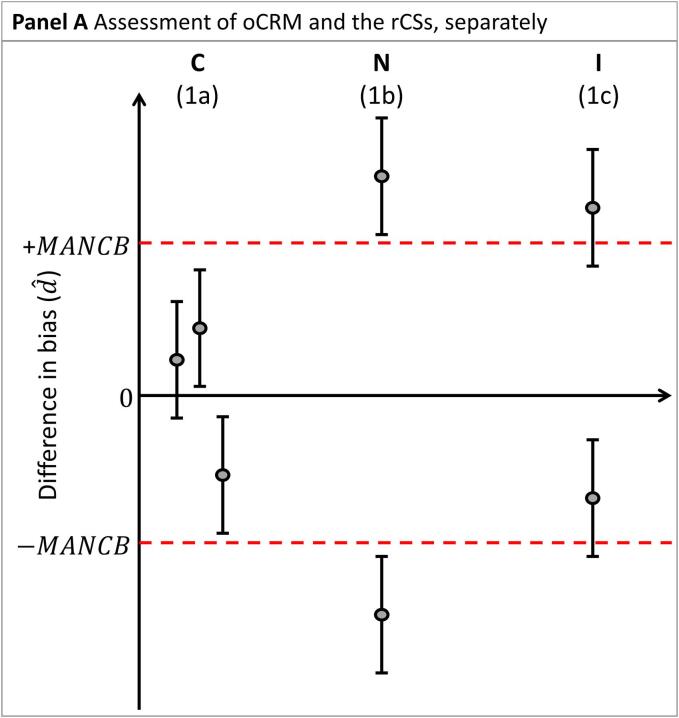

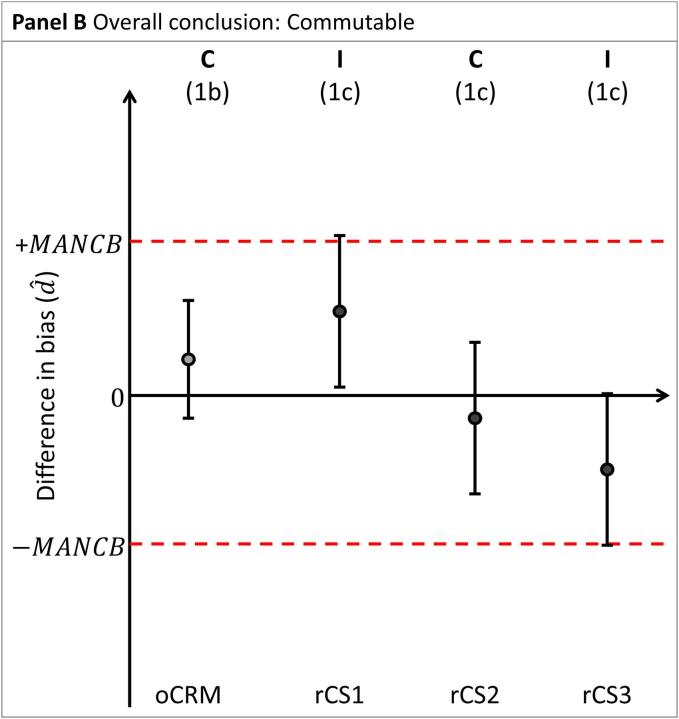

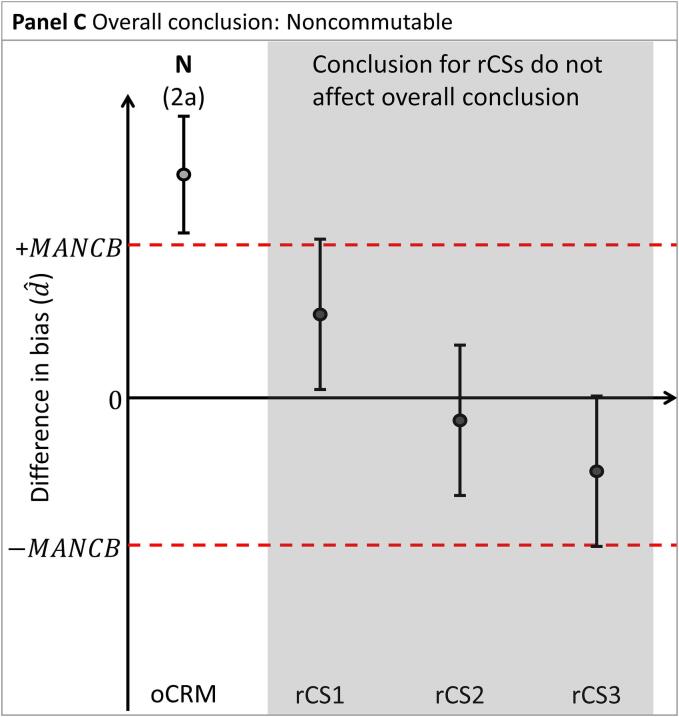

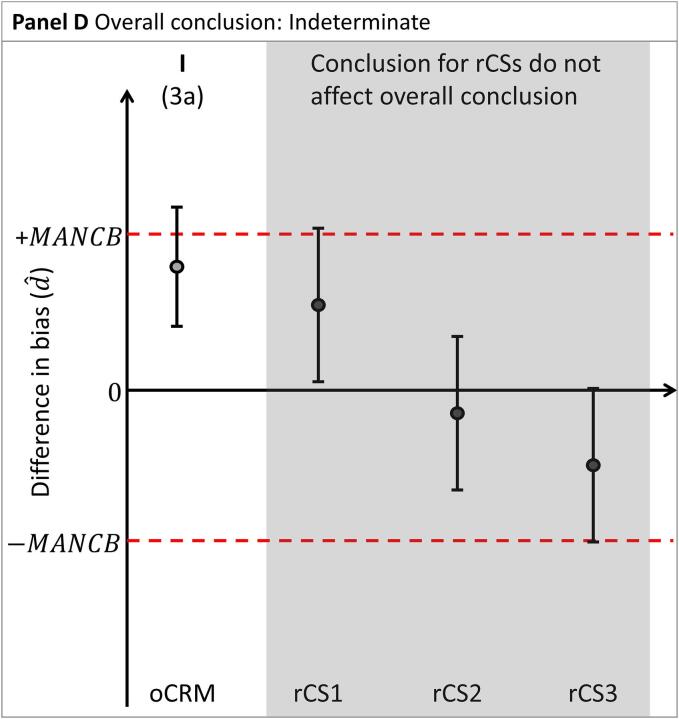

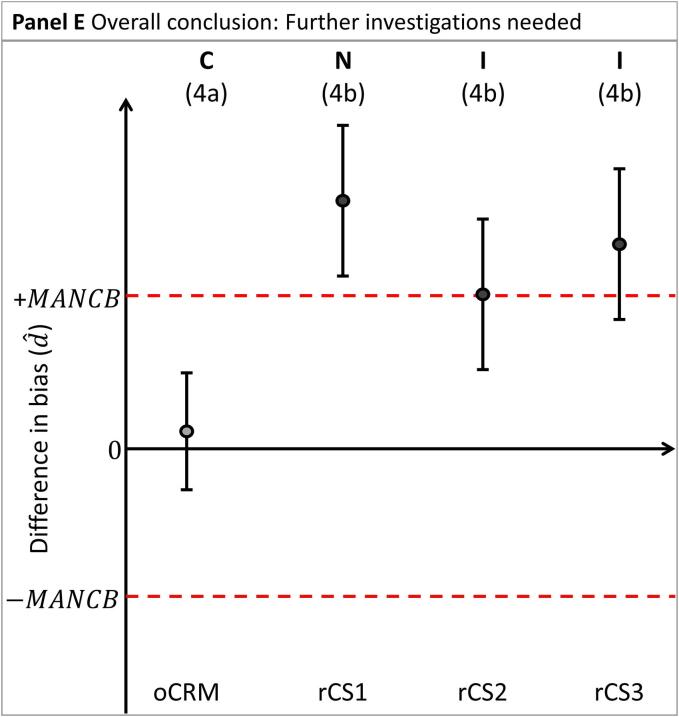
Differences in bias ($\hat{d}$) between two MPs for the nCRM vs. the oCRM, and vs three rCSs (CS1, CS2 and CS3, respectively). Panel A illustrates the assessment of nCRM vs. oCRM and the rCSs, separately. Panels B through E illustrate the four possible overall conclusions for the nCRM. The difference in bias is indicated by shaded circles. Confidence intervals are indicated by vertical whiskers. The MANCB criterion is indicated by horizontal dashed lines. The conclusions are indicated above each material. C: Commutable, N: Noncommutable I: Indeterminate, FIN: Further investigations needed. The corresponding paragraph used for drawing the conclusion is indicated in parenthesis.

To conclude whether the nCRM is commutable, preliminary conclusions are first established for the nCRM relative to each material: the oCRM and the rCSs, separately (see Fig. 2A]:

1. Assessment of nCRM vs the oCRM:
  a. A nCRM is deemed to have the same commutability (designated C in the diagrams) with rCSs as the oCRM when the confidence interval $\hat{d}\pm U\left(\hat{d}\right)$ is entirely within 0 ± MANCB.
  b. A nCRM is deemed not to have the same commutability (designated N in the diagrams) with rCSs as the oCRM when the confidence interval $\hat{d}\pm U\left(\hat{d}\right)$ is entirely outside 0 ± MANCB.
  c. A commutability decision is indeterminate (designated I in the diagrams) when the confidence interval $\hat{d}\pm U\left(\hat{d}\right)$ and 0 ± MANCB are overlapping.
2. Assessment of nCRM against the rCSs:
  a. A nCRM is deemed to be commutable with rCSs (designated C in the diagrams) when the confidence interval $\hat{d}\pm U\left(\hat{d}\right)$ is entirely within 0 ± MANCB.
  b. A nCRM is deemed not to be commutable with rCSs (designated N in the diagrams) when the confidence interval $\hat{d}\pm U\left(\hat{d}\right)$ is entirely outside 0 ± MANCB. In this case, further investigations may be needed to determine the root cause of this observation.
  c. A commutability decision is indeterminate (designated I in the diagrams) when the confidence interval $\hat{d}\pm U\left(\hat{d}\right)$ and 0 ± MANCB are overlapping.

An overall conclusion is obtained by combining the preliminary individual conclusions for the different materials, as described below.

a. The nCRM is concluded to be commutable (Fig. 2B) when:

a. The conclusion for the oCRM was commutable at the time of its production, or in a subsequent assessment, and a proper commutability examination had been done.
b. The conclusion for the nCRM is that it has the same commutability properties as the oCRM.
c. The conclusion for the nCRM is either that it is commutable or indeterminate compared with rCSs.

b. The nCRM is concluded to be noncommutable (Fig. 2C) when:

a. The conclusion for the nCRM is that it does not have the same commutability properties as the oCRM.

c. The nCRM batch is concluded to have indeterminate commutability (Fig. 2D) when:

a. The conclusion for the nCRM is indeterminate compared to the oCRM.

d. A conclusion that further investigations are needed (FIN) (Fig. 2E) when:

e. The conclusion for the nCRM is commutable compared to the oCRM and

f. The conclusion for one or more of the rCSs is noncommutable compared to the nCRM. In this case, further investigations are needed to establish whether the nCRM is commutable.

The overall conclusion for the nCRM is primarily based on its comparison to the oCRM. The conclusions from comparisons to the rCSs must support the conclusion based on nCRM compared to oCRM as a check that the commutability properties of the oCRM have remained stable since originally assessed. Furthermore, the conclusions from the rCSs, when not fully supporting a commutability conclusion for the nCRM, are used to indicate possible areas that warrant further investigation. The confidence intervals $\hat{d}\pm U\left(\hat{d}\right)$ and raw data obtained for the rCSs can be examined for further information.

When further investigation is needed, several situations should be considered. There may be an issue with one or more of the rCSs, e.g., it contains a high amount of an interferent, or pooling has affected its commutability. The oCRM may have unstable commutability properties, causing the oCRM used in the study to be noncommutable at the time of the assessment of the nCRM.

## 6 Conclusion

The recommendations for a commutability equivalence assessment to verify the commutability of a replacement batch of an existing CRM with proven suitable commutability are intended to encourage and facilitate the production of these replacement batches.

However, because many of the currently available CRMs were produced > 10 years ago, conducting a commutability equivalence assessment will not be possible in situations when: i) commutability of the oCRM was not assessed according to the current state of the art, ii) the commutability of the oCRM has not remained stable, or iii) the MPs involved in the original commutability assessment are no longer representative of those in current use in medical laboratories, or their performance has significantly changed since the commutability assessment of the oCRM. However, once a CRM has been assessed for commutability according to current best practices, this commutability equivalence assessment, if applicable within the constraints discussed above, allows for a more economical and practical approach to assessing the commutability of replacement CRM batches.

## CRediT authorship contribution statement

**Liesbet Deprez:** Conceptualization, Writing – original draft. **Jesper V. Johansen:** Conceptualization, Writing – original draft, Methodology. **Thomas Keller:** Conceptualization, Methodology, Writing – review & editing. **Jeffrey Budd:** Conceptualization, Writing – review & editing. **Neil Greenberg:** Conceptualization, Writing – review & editing. **Cas Weykamp:** Conceptualization, Writing – review & editing. **Sverre Sandberg:** Conceptualization, Writing – review & editing. **Mauro Panteghini:** Conceptualization, Writing – review & editing. **Ferruccio Ceriotti:** Conceptualization, Writing – review & editing. **Elizabeth Barczak:** Writing – review & editing. **Robert Rej:** Conceptualization, Writing – review & editing. **Pernille Kjeilen Fauskanger:** Conceptualization, Writing – review & editing. **Finlay MacKenzie:** Conceptualization, Writing – review & editing. **Johanna E. Camara:** Conceptualization, Writing – review & editing. **Alicia N. Lyle:** Conceptualization, Writing – review & editing. **W.Greg Miller:** Conceptualization, Writing – review & editing. **Vincent Delatour:** Conceptualization, Writing – original draft.

## Funding

Pernille K. Fauskanger, Mauro Panteghini and Vincent Delatour have received funding through project 23IND02 COMET of the European Partnership on Metrology, which is co-financed from the European Union’s Horizon Europe Research and Innovation Programme and by the Participating States.

## Declaration of Competing Interest

The authors declare that they have no known competing financial interests or personal relationships that could have appeared to influence the work reported in this paper.

## Data Availability

The data used in the examples are provided in the supplementary data file
