# Supplementary material for: Recommendations for assessing commutability of a replacement batch of a secondary calibrator certified reference material
Source: Clin Chim Acta. 2025 Feb 1;567:120097. doi: 10.1016/j.cca.2024.120097 (PMC11757154; doi:10.1016/j.cca.2024.120097)
Supplement: Supplementary Data 1 [file mmc1.docx]

**Supplementary material to Recommendations for assessing commutability of a replacement batch of a secondary calibrator certified reference material**

**Abbreviations**

CRM: certified reference material

CS : clinical specimen

CI: confidence interval

LCL : lower confidence interval limit

MP: measurement procedure

nCRM : new CRM

oCRM : original CRM

rCS: representative clinical samples

RMP: reference measurement procedure

UCL : upper confidence interval limit

# Examples of commutability equivalence assessments

The following three examples illustrate the process of designing a commutability equivalence assessment, analyzing the obtained data and drawing conclusions.

## Example 1

For the hypothetical measurand M1, the current CRM (designated the original CRM, oCRM) has an assigned value of 150.0 mmol/L and is intended for use as a commutable secondary calibrator in position m.3 in an ISO 17511:2020 calibration hierarchy. The oCRM has been deemed to have acceptable commutability with CSs for eight measurement procedures (MPs) based on a full commutability study using the difference in bias approach [1]. The MANCB criterion was 6.5 mmol/L in the assessment of oCRM and is considered consistent with current clinical use. A replacement batch (designated the new CRM, nCRM) has been manufactured using the same procedures. The estimated M1 concentration of nCRM is 152 mmol/L. This approximate concentration is close enough to the oCRM that the imprecision of MPs is expected to be the same for either batch. Because a reference measurement procedure (RMP) exists, it is included in the assessment. Measurement results from the eight MPs, which remain representative for the current MPs for M1, are compared to the RMP.

The repeatability imprecision estimate for the RMP of 1.7%, corresponding to 2.6 mmol/L at 150 mmol/L, is found in the literature. Hence, the *s_MP1_*/MANCB ratio is 2.6/6.5 = 0.4. From Table 1 (in the main text of this article), using a power of 95%, 10 CRM measurements are needed for each batch of CRM for the RMP. For MPs, repeatability estimates are found in the manufacturer’s IFUs. The number of measurements needed (*n_MPX_*) for the other MPs (MPx) included in the study can be calculated from the imprecisions (*s_MPX_*) of the respective MPs, using the equation (1).

| $n_{MPX}=n_{MP1}\frac{s_{MPX}}{s_{MP1}}$ | (1) |
| --- | --- |

For MP1 through MP6, the repeatability estimates are all close to 3.5%, corresponding to 5.3 mmol/L and this corresponds to $10\cdot\frac{5.3}{2.6}=20.4$, rounded up to 21, measurements needed. For MP7 and MP8, the repeatability estimates are approximately 7.2%, corresponding to 10.9 mmol/L and sample sizes of 42 measurements. Separate test protocols with the higher number of replicates needed for MP7 and MP8 will be followed, rather than using resources conducting larger studies for MP1 through MP6 which only need 21 replicates. The RMP uses the minimum requirement of 10 replicates.

Two CSs and a pool of CSs are used as rCSs. Care has been taken to select CSs without expected interferents and the updated CLSI-C37 protocol [2] was used for their collection. Previous studies have shown that pooling did not affect the commutability of CSs. Enough rCS material has been collected to perform five replicate measurements on the RMP and each of the MPs. The estimated concentrations of the three rCSs are all between approximately 145 and 155 mmol/L. The nCRM and the rCSs all have concentrations close enough that the imprecision of all involved MPs can be assumed approximately constant. This assumption is supported by investigating the scatter of CS results for the original full commutability assessment for the oCRM. Therefore, no transformation of the data is needed.

For the RMP and MP1 the values listed in Table S1 are obtained. For the nCRM, the mean result for the MP1 (150.88 mmol/L) is 0.51 mmol/L higher than the mean result for the RMP (150.37 mmol/L). For the oCRM, the bias is -0.20 mmol/L. The difference in bias is given by:

0.51 mmol/L – (-0.20 mmol/L) = 0.71 mmol/L.

Combining the standard deviations and sample sizes for the two MPs and the two CRM batches according to equation (7) yields a standard uncertainty for the difference in bias ($u\left( \hat{d} \right)$).

$u\left( \hat{d} \right)=\sqrt{\frac{s_{MP1,nCRM}^{2}}{n_{MP1,nCRM}}+\frac{s_{MP2,nCRM}^{2}}{n_{MP2,nCRM}}+\frac{s_{MP1,oCRM}^{2}}{n_{MP1,oCRM}}+\frac{s_{MP2,oCRM}^{2}}{n_{MP2,oCRM}}}$ (7)

where $s$ and $n$ are the sample standard deviations and sample sizes of all measurement results for each MP and material.

$u\left( \hat{d}_{nCRM} \right)=\sqrt{\frac{{3.60}^{2}}{10}+\frac{{4.85}^{2}}{21}+\frac{{2.51}^{2}}{10}+\frac{{3.54}^{2}}{21}}=1.91$ mmol/L.

Multiplying by the coverage factor ($k$) of 1.9 gives the expanded uncertainty $U\left( \hat{d}_{nCRM} \right)$:

$U\left( \hat{d}_{nCRM} \right)$ = $1.91\cdot1.9=3.63$ mmol/L.

The approximate 90% confidence interval (CI) of the difference in bias between nCRM and oCRM is from the lower confidence interval limit (LCL) of (0.71 – 3.63) = -2.91 mmol/L to the upper confidence interval limit (UCL) of (0.71 +3.63) = 4.34 mmol/L. Because this interval contains no values numerically larger than the MANCB of 6.5 mmol/L, the nCRM is deemed commutable, ie, it has commutability properties equivalent to those of the oCRM.

The calculations for the nCRM compared to the rCSs are performed the same way.

The calculated differences in bias and the associated CIs for MP1 are plotted in Figure S1 below. The estimated concentration of the nCRM is indicated by a blue square. There is no difference in bias possible between the value for nCRM and itself, so the point is at zero on the y-axis with no CI possible.

**Table S1:** Calculation summary for commutability equivalence assessment of nCRM in example 1 for the MP comparison MP1 vs the RMP

| **Material** | **RMP** | | | **MP1** | | | $\boldsymbol{b}$  **x_MP1_ - x_RMP_** | $\hat{\boldsymbol{d}}$  $\boldsymbol{b}_{\boldsymbol{nCRM}}\boldsymbol{-}\boldsymbol{b}_{\boldsymbol{Material}}$ | $\boldsymbol{u(}\hat{\boldsymbol{d}}\boldsymbol{)}$ | $\boldsymbol{k}$ | $\boldsymbol{U(}\hat{\boldsymbol{d}}\boldsymbol{)}$ | **LCL** | **UCL** | **Conclusion** |
| --- | --- | --- | --- | --- | --- | --- | --- | --- | --- | --- | --- | --- | --- | --- |
|  | **n** | **Mean** | ***s*** | **n** | **Mean** | ***s*** |  |  |  |  |  |  |  |  |
| **nCRM** | 10 | 150.37 | 3.60 | 21 | 150.88 | 4.85 | 0.51 |  |  |  |  |  |  |  |
| **oCRM** | 10 | 152.03 | 2.51 | 21 | 151.83 | 3.54 | -0.20 | 0.71 | 1.91 | 1.9 | 3.63 | -2.91 | 4.34 | Commutable |
| **CS1** | 5 | 147.72 | 2.52 | 5 | 148.32 | 1.41 | 0.60 | -0.09 | 2.02 | 2.3 | 4.65 | -4.74 | 4.56 | Commutable |
| **CS2** | 5 | 154.00 | 2.03 | 5 | 150.62 | 4.91 | -3.38 | 3.89 | 2.84 | 2.3 | 6.53 | -2.64 | 10.42 | Indeterminate |
| **Pool** | 5 | 148.72 | 2.35 | 5 | 151.46 | 2.87 | 2.74 | -2.23 | 2.27 | 2.3 | 5.23 | -7.46 | 3.00 | Indeterminate |


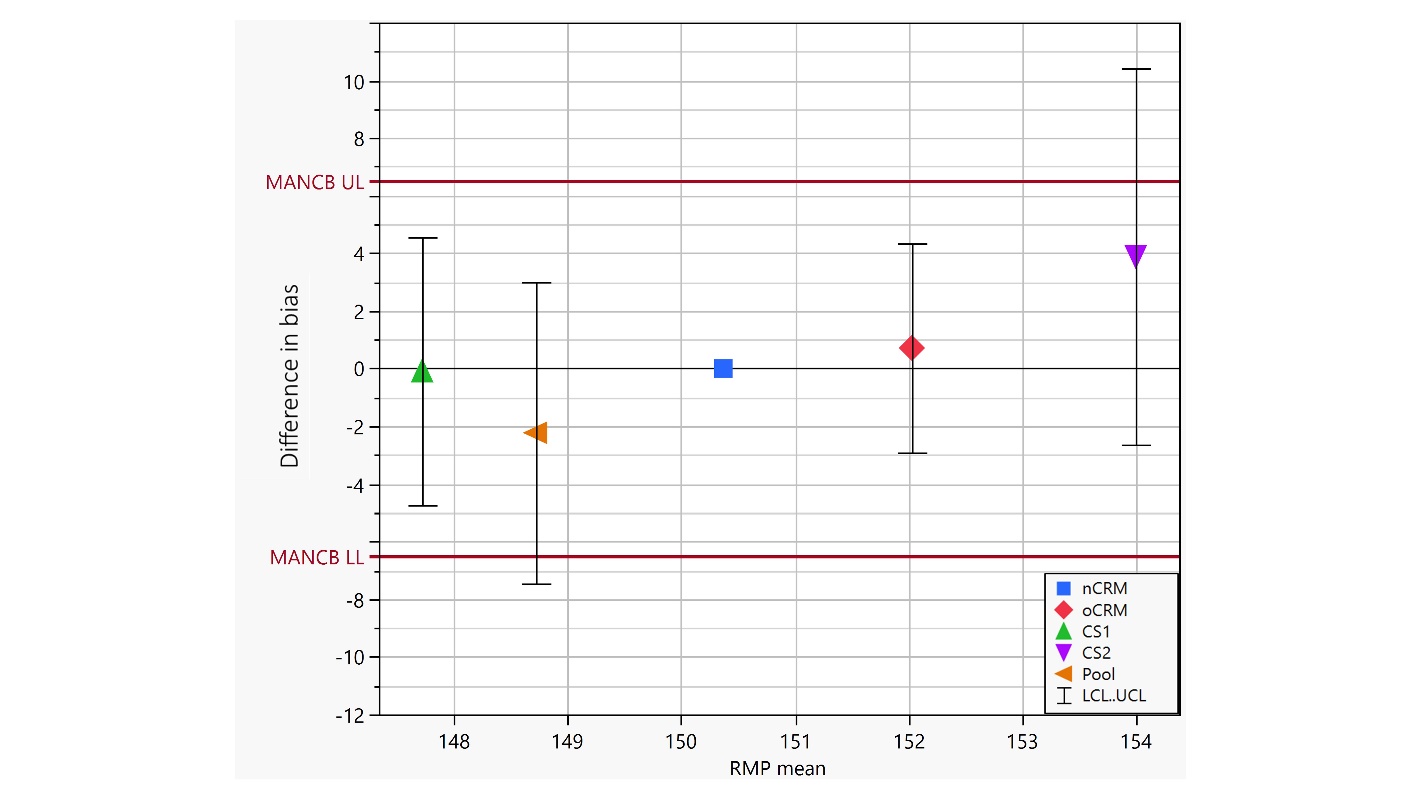


**Figure S1**: Graphical illustration of commutability equivalence assessment of nCRM in example 1 for the MP comparison MP1 vs the RMP. The graph shows the differences in bias ($\hat{d}$) between MP1 and the RMP for the nCRM vs the oCRM, and vs the rCSs (the two CSs and one CS pool) as function of the concentration estimated using the RMP. CIs are indicated by vertical whiskers. The MANCB criterion is indicated by horizontal lines. The concentration of the nCRM estimated using the RMP is indicated by a blue square.

The process was repeated for the seven other MPs paired with the RMP. The results are summarized in table S2 below.

**Table S2:** Calculation summary for commutability equivalence assessment of nCRM in example 1 for the eight MP comparisons (MPx vs the RMP)

|  | **MP1** | **MP2** | **MP3** | **MP4** | **MP5** | **MP6** | **MP7** | **MP8** |
| --- | --- | --- | --- | --- | --- | --- | --- | --- |
| **oCRM** | C | I | C | C | C | C | C | C |
| **CS1** | C | C | C | I | I | N | I | I |
| **CS2** | I | I | C | I | I | I | I | I |
| **Pool** | I | I | I | I | C | N | I | I |

C: commutable, I: indeterminate, N: non-commutable

**Table S3**: Overall conclusion of the commutability equivalence assessment for nCRM in example 1

|  | **MP1** | **MP2** | **MP3** | **MP4** | **MP5** | **MP6** | **MP7** | **MP8** |
| --- | --- | --- | --- | --- | --- | --- | --- | --- |
| **oCRM** | C | I | C | C | C | C | C | C |
| **rCSs** | OK | OK | OK | OK | OK | Not OK | OK | OK |
| **Conclusion** | Commu-table | Indeter-minate | Commu-table | Commu-table | Commu-table | Further investi-gations needed | Commu-table | Commu-table |

The nCRM is deemed commutable with CSs for use with MPs 1, 3, 4, 5, 7, and 8 because the conclusion is commutable for the nCRM vs the oCRM and the conclusion is NOT noncommutable for nCRM vs all rCSs for these MPs. For MP2, the conclusion is indeterminate because the CI for the difference in bias for the nCRM overlaps the MANCB.

For MP6, because the CI of the difference in bias for the nCRM vs oCRM is entirely within the interval [-MANCB; MANCB], it is concluded that the nCRM has commutability properties equivalent to those of the oCRM. However, because the nCRM is deemed noncommutable with the CS1 and pool rCSs, there is an indication of issues with these rCSs, the nCRM or the commutability equivalence assessment. Therefore, further investigations are needed into why the nCRM is deemed noncommutable with CS1 and the pool when measured using MP6. It is always important to investigate possible lab errors or sample mix-up.

Figure S3 shows the results for MP6. Different hypothesis can be formulated. A possible explanation is that the pool is not a representative CS in the sense that it contains an unexpected interfering substance or the pooling process has somehow altered the matrix. Because CS1 and the pool gave a noncommutable conclusion only for MP6 vs. the RMP, it can also be hypothesized that MP6 has poorer selectivity for the measurand than the other MPs. Looking at data of the original full commutability study done for oCRM can help confirm if pairwise comparisons involving MP6 had larger sample specific effects than those involving other MPs.


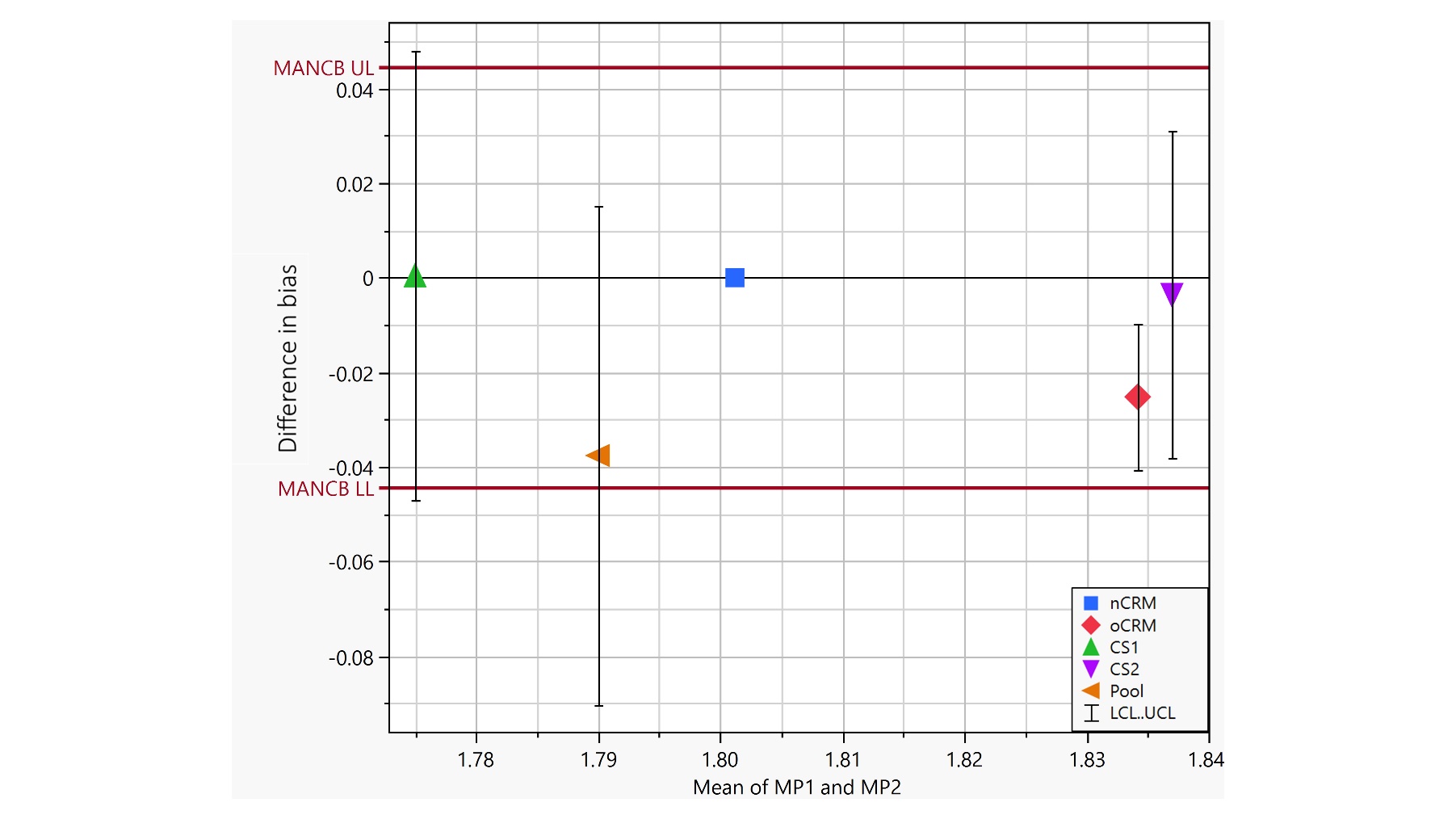


**Figure S2:** Graphical illustration of commutability equivalence assessment of nCRM in example 1 for the MP comparison MP6 vs the RMP. The graph shows differences in bias ($\hat{d}$) between MP6 and the RMP for the oCRM, and the rCSs (the two CSs and one CS pool) vs the nCRM as function of the concentration estimated using the RMP. CIs are indicated by vertical whiskers. The MANCB criterion is indicated by horizontal lines. The concentration of the nCRM estimated using the RMP is indicated by a blue square.

The commutability of the oCRM compared to CS1 and the pool might give additional insight into the nature of the issue. For MP6, the bias is -1.44 mmol/L and 0.73 mmol/L for the nCRM and oCRM, respectively, and 14.40 mmol/L, 10.06 mmol/L, and 10.88 mmol/L for CS1, CS2 and the pool, respectively. Even without calculating the difference in bias and the corresponding CIs, it is evident that all rCSs behave very different from the CRMs when comparing MP6 to the RMP. Because all three rCSs have a negative difference in bias for MP6, this could be an indication that the selectivity of MP6 has changed since the commutability study on the oCRM was conducted. Because the nCRM is designed to resemble the oCRM as much as possible, any bias for these two CRMs should be identical for both MPs, resulting in a difference in bias near zero. However, the CRMs may differ in matrix from the rCSs. CSs are commutable by definition assuming selectivity of an MP is acceptable and specimen specific influences are absent. Hence, the results for the rCSs could indicate that neither the oCRM nor the nCRM are commutable for MP6 in its current configuration. Further investigations are needed to resolve this issue. If it is concluded that the selectivity of MP6 has changed since the commutability study for the oCRM, MP6 may be excluded. The IVD provider should be consulted to identify possible changes in the reagent formulation and/or other characteristics of MP6.

Regardless of these considerations for the pool measured using MP6, the overall conclusion is that the commutability of the nCRM with CSs is equivalent to the commutability of the oCRM when used with all MPs except MP6. As stated in the overall conclusion table above, further investigations are needed before making a conclusion regarding the commutability of nCRM with CSs when used with MP6.

## Example 2

For the hypothetical measurand M2, the current CRM (oCRM) is intended for use as a commutable secondary calibrator in position m.3 in an ISO 17511:2020 calibration hierarchy. This oCRM has an assigned value of 1.81 mmol/L. The oCRM has been deemed commutable with CSs based on a recent full commutability study (based on the difference in bias approach) with seven MPs using the MANCB criterion of 0.044 mmol/L. This criterion is considered consistent with current clinical use. A replacement batch (nCRM) has been manufactured using the same procedures. The estimated M2 concentration of nCRM is 1.83 mmol/L. This approximate concentration is close enough to the oCRM that the imprecision of all MPs is expected to be the same for measuring either CRM. Because no RMP exists, only end-user MPs are included in the assessment. Measurement results from the seven MPs are all compared to each other.

Following the approach shown in Example 1, 25 replicate measurements are needed for each batch of CRM. Two CSs and a pool of CSs are used as rCSs. Care has been taken to select CSs without expected interferents. Previous studies have shown that pooling did not affect the commutability of CSs. Enough rCS material has been collected to perform five replicate measurements on the each of the MPs. The estimated concentrations of the three rCSs are all between 1.78 and 1.85 mmol/L. The nCRM and the rCSs all have concentrations close enough that the imprecision for measuring nCRM and rCSs can be assumed approximately the same for all samples, within each MP in the assessment. This assumption is supported by investigation of the scatter of CS results for the original full commutability assessment for the oCRM. Therefore, no transformation of the data is needed. The calculation results for MP1 compared to MP2 for the nCRM, oCRM and the rCSs are summarized in Table S4. The calculated differences in bias and the associated CIs are plotted in figure S3 below.

**Table S4:** Calculation summary for commutability equivalence assessment of nCRM in example 2 for the MP comparison MP2 vs the MP1.

| **Material** | **MP1** | | | **MP2** | | | $\boldsymbol{b}$  **x_MP2_ – x_MP1_** | $\hat{\boldsymbol{d}}$  $\boldsymbol{b}_{\boldsymbol{nCRM}}\boldsymbol{-}\boldsymbol{b}_{\boldsymbol{Material}}$ | $\boldsymbol{u(}\hat{\boldsymbol{d}}\boldsymbol{)}$ | $\boldsymbol{k}$ | $\boldsymbol{U(}\hat{\boldsymbol{d}}\boldsymbol{)}$ | **LCL** | **UCL** | **Conclusion** |
| --- | --- | --- | --- | --- | --- | --- | --- | --- | --- | --- | --- | --- | --- | --- |
|  | **n** | **Mean** | ***s*** | **n** | **Mean** | ***s*** |  |  |  |  |  |  |  |  |
| **nCRM** | 25 | 1.808 | 0.013 | 25 | 1.794 | 0.023 | -0.014 |  |  |  |  |  |  |  |
| **oCRM** | 25 | 1.828 | 0.013 | 25 | 1.840 | 0.028 | 0.012 | -0.025 | 0.008 | 1.9 | 0.015 | -0.041 | -0.010 | Commutable |
| **CS1** | 5 | 1.782 | 0.028 | 5 | 1.768 | 0.035 | -0.014 | 0.000 | 0.021 | 2.3 | 0.047 | -0.047 | 0.048 | Indeterminate |
| **CS2** | 5 | 1.842 | 0.015 | 5 | 1.832 | 0.028 | -0.010 | -0.004 | 0.015 | 2.3 | 0.035 | -0.038 | 0.031 | Commutable |
| **Pool** | 5 | 1.778 | 0.015 | 5 | 1.802 | 0.048 | 0.024 | -0.038 | 0.023 | 2.3 | 0.053 | -0.090 | 0.015 | Indeterminate |

**Figure S3:** Graphical illustration of commutability equivalence assessment of nCRM in example 2 for the MP comparison MP2 vs the MP1. The graph shows differences in bias ($\hat{d}$) for MP2 compared to MP1 for the oCRM, and the rCSs (the two CSs and one CS pool) as function of the mean concentration estimated using MP1 and MP2. CIs are indicated by vertical whiskers. The MANCB criterion is indicated by horizontal lines. The concentration of the nCRM estimated using MP1 is indicated by a blue square.


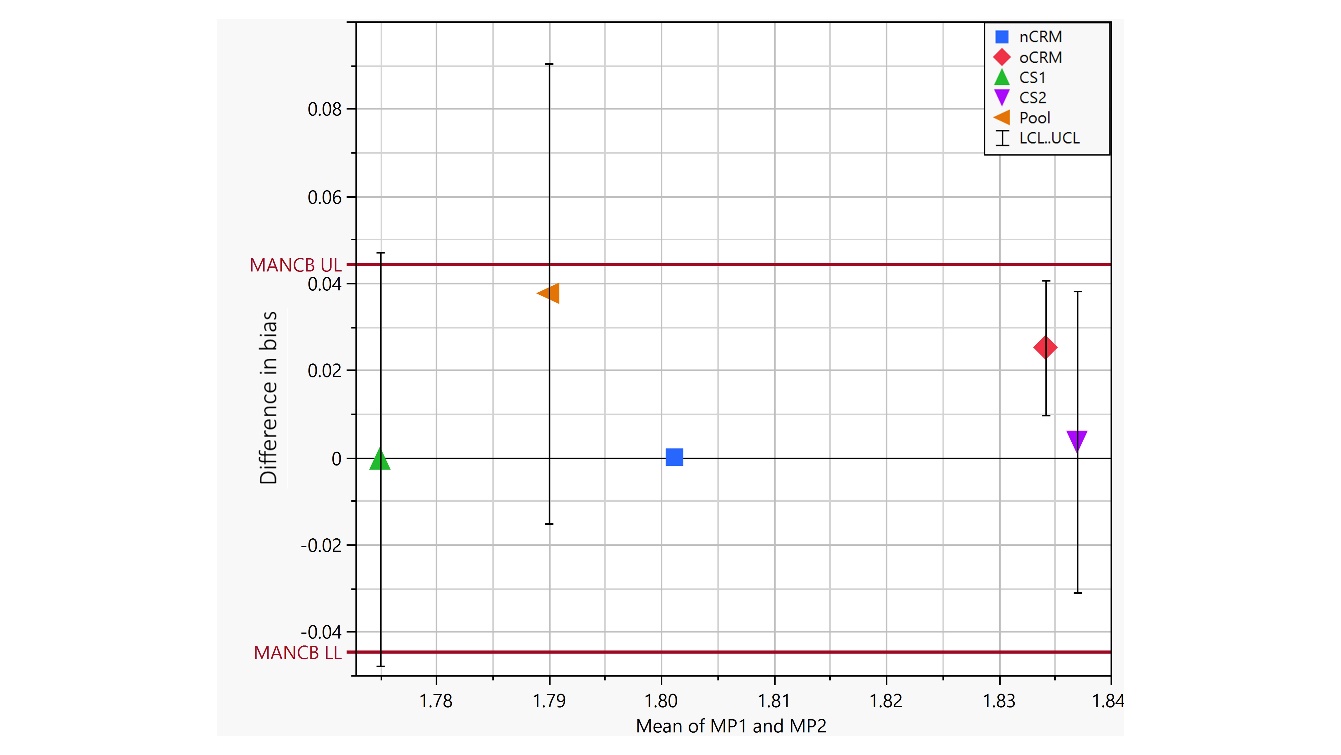


Because the CI of the difference in bias for the nCRM vs the oCRM is entirely within the interval [-MANCB; MANCB], the nCRM is deemed to have equivalent properties as the oCRM indicated by C in the table. Because nCRM vs rCSs does not have any noncommutable conclusions, there is no indication of issues with the oCRM or the commutability equivalence assessment. The process was repeated for the other MPs pairs. The results are summarized in table S5 and S6.

**Table S5:** Calculation summary for commutability equivalence assessment of nCRM in example 2 for the 21 MP pairs (MPx vs MPx)

| **MP** | **Material** | **Conclusion for nCRM vs the indicated material for each MP pair** | | | | | |
| --- | --- | --- | --- | --- | --- | --- | --- |
|  |  | **MP2** | **MP3** | **MP4** | **MP5** | **MP6** | **MP7** |
| **MP1** | oCRM | C | C | C | C | C | C |
| **MP1** | CS1 | I | I | I | I | I | I |
| **MP1** | CS2 | C | C | C | C | C | I |
| **MP1** | Pool | I | I | I | I | I | I |
| **MP2** | oCRM |  | C | C | I | C | I |
| **MP2** | CS1 |  | I | I | I | I | I |
| **MP2** | CS2 |  | I | C | I | C | I |
| **MP2** | Pool |  | I | I | I | I | I |
| **MP3** | oCRM |  |  | C | C | C | C |
| **MP3** | CS1 |  |  | I | I | I | I |
| **MP3** | CS2 |  |  | C | I | C | I |
| **MP3** | Pool |  |  | I | I | I | I |
| **MP4** | oCRM |  |  |  | C | C | C |
| **MP4** | CS1 |  |  |  | I | I | I |
| **MP4** | CS2 |  |  |  | I | C | I |
| **MP4** | Pool |  |  |  | I | I | I |
| **MP5** | oCRM |  |  |  |  | C | C |
| **MP5** | CS1 |  |  |  |  | I | I |
| **MP5** | CS2 |  |  |  |  | I | I |
| **MP5** | Pool |  |  |  |  | I | I |
| **MP6** | oCRM |  |  |  |  |  | C |
| **MP6** | CS1 |  |  |  |  |  | I |
| **MP6** | CS2 |  |  |  |  |  | I |
| **MP6** | Pool |  |  |  |  |  | I |

C: commutable, I: indeterminate, N: non-commutable

**Table S6**: Overall conclusion of the commutability equivalence assessment for nCRM in example 2

|  | **Overall conclusion of the commutability equivalence assessment for nCRM for each MP pair** | | | | | |
| --- | --- | --- | --- | --- | --- | --- |
| **MP** | **MP2** | **MP3** | **MP4** | **MP5** | **MP6** | **MP7** |
| **MP1** | C | C | C | C | C | C |
| **MP2** |  | C | C | I | C | I |
| **MP3** |  |  | C | C | C | C |
| **MP4** |  |  |  | C | C | C |
| **MP5** |  |  |  |  | C | C |
| **MP6** |  |  |  |  |  | C |

C: commutable, I: indeterminate, N: non-commutable

This example highlights that data interpretation requires measurement results from all MPs to be compared to each other when no RMP is available, therefore decision making is more complex. Pairwise comparisons for nCRM vs oCRM had indeterminate results for both MP5 and MP7 compared to MP2. Consequently, the nCRM was deemed commutable with CSs when used with all MPs except MP2. The reason for the indeterminate conclusions for MP2 should be investigated. Possible explanations include: i) insufficient precision of MP2, which can be verified using data of the commutability equivalence assessment and from the original commutability study for oCRM; ii) insufficient and/or differences in non-selectivity of MP2 compared to other MPs, which can be evaluated using data of the original commutability study for oCRM; and iii) error in handling, labelling, or reporting. A large number of indeterminate conclusions were reached for the rCS, which likely reflects the lower number of replications of measurements for these samples. Since there were no noncommutable conclusions from the rCS measurements, the data is supportive that the commutability conclusions for the nCRM are valid.

## Example 3

For the hypothetical measurand M3, the conditions and study design are identical to those described in Example 2. Table S7 summarizes the conclusions for all MP pairs and the overall conclusions are summarized in table S8. A conclusion of indeterminate (I) or noncommutable (N) indicates that there is likely a problem with the nCRM. In these scenarios, the investigation should address issues with the new batch of CRM. The abbreviation FIN indicates that further investigation is needed because a conclusion of commutable for the nCRM is not supported by the rCSs of which at least one was found noncommutable. Hence, a conclusion of FIN indicates that there is likely a problem with the study, the selected rCSs, or with the oCRM. In this scenario, the investigation should address issues with the commutability equivalence assessment, including potential issues with the selected rCSs. Several types of issues are identified in this study.

**Table S7:** Calculation summary for commutability equivalence assessment of nCRM in example 3 for the 21 MP pairs (MPx vs MPx)

|  |  | **Conclusion for nCRM vs the indicated material for each MP pair** | | | | | |
| --- | --- | --- | --- | --- | --- | --- | --- |
| **MP** | **Material** | **MP2** | **MP3** | **MP4** | **MP5** | **MP6** | **MP7** |
| **MP1** | oCRM | I | I | I | I | C | N |
| **MP1** | CS1 | N | I | N | I | N | N |
| **MP1** | CS2 | N | I | I | I | I | N |
| **MP1** | Pool | I | N | I | I | N | I |
| **MP2** | oCRM |  | C | C | C | I | I |
| **MP2** | CS1 |  | I | I | I | I | I |
| **MP2** | CS2 |  | N | I | I | N | I |
| **MP2** | Pool |  | I | N | I | I | I |
| **MP3** | oCRM |  |  | C | I | I | I |
| **MP3** | CS1 |  |  | I | I | I | N |
| **MP3** | CS2 |  |  | I | I | I | N |
| **MP3** | Pool |  |  | N | I | I | I |
| **MP4** | oCRM |  |  |  | C | I | I |
| **MP4** | CS1 |  |  |  | I | I | I |
| **MP4** | CS2 |  |  |  | I | I | N |
| **MP4** | Pool |  |  |  | N | N | N |
| **MP5** | oCRM |  |  |  |  | I | I |
| **MP5** | CS1 |  |  |  |  | I | I |
| **MP5** | CS2 |  |  |  |  | I | N |
| **MP5** | Pool |  |  |  |  | I | I |
| **MP6** | oCRM |  |  |  |  |  | N |
| **MP6** | CS1 |  |  |  |  |  | I |
| **MP6** | CS2 |  |  |  |  |  | N |
| **MP6** | Pool |  |  |  |  |  | I |

C: commutable, I: indeterminate, N: non-commutable

**Table S8**: Overall conclusion of the commutability equivalence assessment for nCRM in example 3

|  | **Overall conclusion of the commutability equivalence assessment for nCRM for each MP pair** | | | | | |
| --- | --- | --- | --- | --- | --- | --- |
| **MP** | **MP2** | **MP3** | **MP4** | **MP5** | **MP6** | **MP7** |
| **MP1** | I | I | I | I | FIN | N |
| **MP2** |  | FIN | FIN | C | I | I |
| **MP3** |  |  | FIN | I | I | I |
| **MP4** |  |  |  | FIN | I | I |
| **MP5** |  |  |  |  | I | I |
| **MP6** |  |  |  |  |  | N |

C: commutable, I: indeterminate, N: non-commutable, FIN: Further investigations needed

The nCRM was found to have the same commutability property with CSs as the oCRM for only 1 MP pair, noncommutable for 2 MP pairs, indeterminate for 13 of the 21 MP pairs, and further investigations are needed for 9 of the 21 MP pairs. Furthermore, conclusions based on the rCSs are noncommutable in 19 of 63 pairings. Hence, the commutability issues identified for the nCRM are also observed for the rCSs. For most of the MP pairs where the nCRM was found to be either noncommutable or indeterminate with the oCRM, noncommutable conclusions were also reached based on one or more of the rCSs. It is likely that the commutability properties of the nCRM and oCRM are different for the MPs in the assessment. The differences in commutability are likely due to differences in the preparation of the nCRM, which requires further investigation. The issues might also be caused by differences in non-selectivity of the MPs combined with sample specific effects in the rCSs and possibly in the nCRM. Collectively, the study results do not support that the nCRM is suitable for use. A full commutability study for the nCRM was not pursued because the commutability equivalence assessment does not support commutability of the nCRM. A more productive follow up would be to discard the nCRM and produce a new batch after investigating the likely cause(s) of the noncommutability. Furthermore, if one or more MPs are confirmed to have unacceptable selectivity for the measurand, that MP would be excluded from a subsequent commutability assessment.

# Checklist of requirements for conducting a commutability equivalence assessment and analyzing its outcome

CRM producers can use the following checklist to have a clear overview of all of the requirements. In addition, this checklist can be used as supporting information for JCTLM submission of CRM replacement batches or for scientific publications.

| **Requirements for the oCRM** | **Yes/No/Partly** | **Conclusion^1^** |
| --- | --- | --- |
| Was commutability of the oCRM evaluated according to the most recent recommendations and statistical approaches? If not, were data reanalyzed using the most recent recommendations and statistical approaches? |  |  |
| Did the acceptance criterion match the intended use of the nCRM? If not, were data reanalyzed with a suitable commutability criterion? |  |  |
| Were the number of replicate measurements performed on the oCRM and the CSs sufficient for providing an acceptable estimate of the uncertainty associated with the commutability assessment? |  |  |
| Are the MPs involved in the commutability assessment of the oCRM adequately representative of those in current use in medical laboratories? Did their performance remain unchanged since the assessment of the oCRM? |  |  |
| Is there evidence supporting that the oCRM has maintained the same commutability properties since the time that the measurements for the commutability study were performed? |  |  |
| Is the oCRM available in sufficient amount to allow measuring the required number of replicates on all relevant MPs? |  |  |
| **Requirements for the nCRM** |  |  |
| Are the concentrations of the measurand in the nCRM and oCRM sufficiently close that the imprecision of measuring the oCRM and nCRM is the same? |  |  |
| Is the nCRM intended and expected to have the same commutability properties as the oCRM, ie, are the specifications for preparation, production process and raw materials of the nCRM equivalent to those from the oCRM? |  |  |

| Requirements for the representative clinical samples (rCSs) | **Yes/No/Partly** | **Conclusion^1^** |
| --- | --- | --- |
| Does the commutability equivalence assessment include a minimum of three rCSs? |  |  |
| Are the rCSs selected to avoid samples with large amounts of interfering substances to the extent possible? |  |  |
| If pools are used, were the procedures used during the pool production (e.g. longer storage periods, additional freeze thaw cycles or mixing) tested to not affect their commutability compared to individual CS? |  |  |
| Design of the commutability equivalence assessment |  |  |
| Was the maximum allowable noncommutability bias (MANCB), established following the latest recommendations? |  |  |
| Are the IVD-MPs included in the commutability equivalence assessment adequately representative of those in current use in medical laboratories? |  |  |
| If an RMP is available, is it included in the commutability equivalence assessment? |  |  |
| Were the nCRM, the oCRM and the rCSs measured with each MP using the same reagent and calibrator lots? |  |  |
| Were all measurements for the nCRM, the oCRM and the rCSs measured within a single run on each MP? |  |  |
| Outcome of the commutability equivalence assessment |  |  |
| In the assessment of nCRM vs the oCRM, was the nCRM concluded to have equivalent commutability to the oCRM (conclusion: Commutable, C)? |  |  |
| In the assessment of nCRM against the rCSs, does the conclusion for any of the rCSs warrant further investigations (conclusion for one or more rCSs: Noncommutable, N)? |  |  |

In case of ”Partly”, assess whether the requirement is fulfilled to a degree that a full commutability assessment is not required. Consider the intended use and potential clinical impact of the measurand(s).

**References**

1. Nilsson G, Budd JR, Greenberg N, Delatour V, Rej R, Panteghini M, et al. IFCC working group recommendations for assessing commutability part 2: using the difference in bias between a reference material and clinical samples. Clin Chem 2018;64:455–64.
2. Danilenko U, Vesper HW, Meyers GL, Clapshaw PA, Camara JE, Miller GW. An updated protocol based on CLSI document C37 for preparation of off-the-clot serum from individual units for use alone or to prepare commutable pooled serum reference materials. Clin Chem Lab Med, 2020; 58:368-74.
